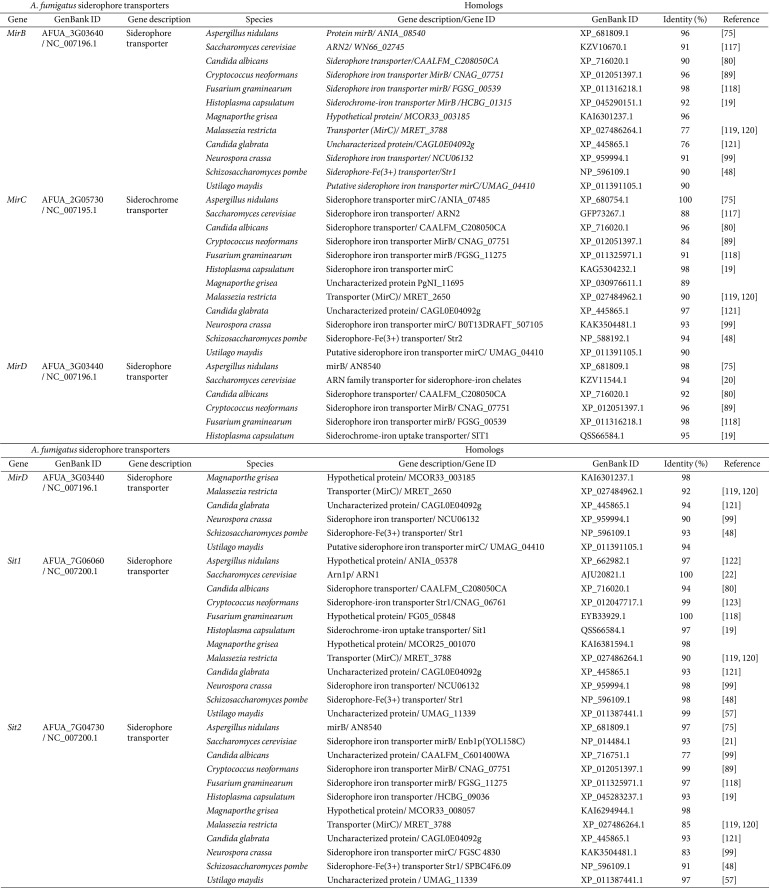# Siderophore Biosynthesis and Transport Systems in Model and Pathogenic Fungi

**DOI:** 10.4014/jmb.2405.05020

**Published:** 2024-06-13

**Authors:** Sohyeong Choi, James W. Kronstad, Won Hee Jung

**Affiliations:** 1 Department of Systems Biotechnology, Chung-Ang University, Anseong 17546, Republic of Korea; 2 Michael Smith Laboratories, Department of Microbiology and Immunology, University of British Columbia, Vancouver, British Columbia, Canada

**Keywords:** Fungus, iron, siderophore, virulence

## Abstract

Fungi employ diverse mechanisms for iron uptake to ensure proliferation and survival in iron-limited environments. Siderophores are secondary metabolite small molecules with a high affinity specifically for ferric iron; these molecules play an essential role in iron acquisition in fungi and significantly influence fungal physiology and virulence. Fungal siderophores, which are primarily hydroxamate types, are synthesized via non-ribosomal peptide synthetases (NRPS) or NRPS-independent pathways. Following synthesis, siderophores are excreted, chelate iron, and are transported into the cell by specific cell membrane transporters. In several human pathogenic fungi, siderophores are pivotal for virulence, as inhibition of their synthesis or transport significantly reduces disease in murine models of infection. This review briefly highlights siderophore biosynthesis and transport mechanisms in fungal pathogens as well the model fungi *Saccharomyces cerevisiae* and *Schizosaccharomyces pombe*. Understanding siderophore biosynthesis and transport in pathogenic fungi provides valuable insights into fungal biology and illuminates potential therapeutic targets for combating fungal infections.

## Introduction

Iron is an essential element for all organisms because it serves as a cofactor in numerous enzymes for various cellular processes. Microbes, including pathogenic fungi, have developed a variety of strategies for acquiring iron and these include 1) reductive iron uptake (reduction of ferric iron to ferrous iron), 2) extraction and capture of iron from host sources such as heme, and 3) siderophore-mediated iron uptake.

Siderophores are secondary metabolites with a molecular weight of 200 to 2,000 Da that are secreted mainly by microbes and plants, and possess a high affinity specifically for ferric iron [1]. Most microbes produce their own siderophores to efficiently uptake iron under iron-deficient environments and, in some cases, they can also use siderophores synthesized by other organisms (so called xenosiderophores). Microbial siderophores are typically synthesized in response to iron depletion, and it has been estimated that at least 500 different types of siderophores have been classified from multiple organisms [1]. Siderophores can be classified into five main types: hydroxamates, catecholates, carboxylates, phenolates, and mixed-types (Table 1) [2]. Hydroxamate siderophores are made up of acylated and hydroxylated alkylamine in bacteria and hydroxylated and alkylated ornithine in fungi. Almost all fungal siderophores are hydroxamate types, which are further grouped into three categories: fusarinines, coprogens, and ferrichromes [3]. The first fungal hydroxamate siderophores, coprogen and ferrichrome, were identified from the smut fungus *Ustilago sphaerogana* in 1952 [4, 5]. Catecholate siderophores contain carboxyl and hydroxyl groups, and are normally found in bacteria as first discovered in *Bacillus subtilis* grown in low iron conditions [6, 7]. However, catecholate siderophores are also considered one of the major types of fungal siderophores produced by zygomycetes including, for example, rhizoferrin produced from *Rhizopus microspore* [8, 9]. Phenolate siderophores are mainly produced from bacteria, such as enterobactins from *Escherichia coli* and *Salmonella typhimurium* [10]. Mixed-type siderophores structurally correspond to more than one different types of siderophores. For example, heterobactin synthesized by *Rhosococcus erythropolis* is composed of both catecholate and hydroxamate groups [11]. Lysine derivatives such as myobactin, and ornithine derivatives such as pyoverdine, also belong to the mixed-type siderophores [12].

Siderophore biosynthesis is mediated by two different pathways. The first involves non-ribosomal peptide synthetase (NRPS), a multimodule enzyme complex responsible for generating structurally highly variable peptides without the use of an RNA template. The second is the NRPS-independent pathway, which is carried out by several enzymes including monooxygenases, decarboxylases, amino and acetyltransferases, amino acid ligases, and aldolases to assemble siderophores. Most hydroxamate and carboxylate siderophores are synthesized by NRPS-independent pathway [1]. After synthesis, siderophores are secreted and chelate iron in the extracellular environment. Iron-bound siderophore is then transported through the cell membrane via a few different mechanisms. In Gram-negative bacteria, the TonB-ExbB-ExbD transport protein complex at the outer membrane is responsible for siderophore uptake from the environment. Subsequently, iron-bound siderophores are further transported by permeases or ATP-binding cassette (ABC) transporters at the cytoplasmic membrane to the cytoplasm. Gram-positive bacteria do not possess the TonB-dependent transporter, but instead utilize ABC transporters in the plasma membrane. Subsequently, iron is dissociated from siderophores by reductive processes involved in the ferric to ferrous iron transition [13]. Several different siderophore uptake mechanisms in fungi have been identified, and these are thoroughly summarized by Das *et al*. [14]. Among them, a shuttle mechanism is responsible for the uptake of most common fungal siderophores including ferrichrome and coprogen types, and is involved in the transport of the iron-bound siderophore by a siderophore-specific membrane transporter. After internalization, the release of iron from iron-bound siderophores occurs either by a siderophore-specific reductase or by direct ligand exchange. In the case of triacetylfusarinine C, which is the major hydroxamate-type siderophore produced in *Aspergillus fumigatus*, an esterase mediates iron release by cleaving the ester bonds of the iron-bound siderophore after uptake [15]. In addition, the involvement of cell surface reduction by reductases for the uptake of iron-bound siderophores in fungi has been reported. For example, the activity of plasma membrane reductases, the Fre proteins, was shown to be required for the uptake of ferrioxamine B, ferrichrome, and triacetylfusarinine C in the model fungus *Saccharomyces cerevisiae* [16].

Iron is essential for the virulence of fungal pathogens during infection, and the competition for iron between the fungal pathogens and the host significantly influences the disease processes. Fungi have developed several sophisticated strategies, including the use of siderophores, for iron acquisition under the low-iron condition of the host. Siderophores play critical roles in virulence for many but not all fungal pathogens of humans. For example, in *A. fumigatus*, the lack of either siderophore biosynthesis or the transport system significantly reduces virulence in the mouse infection model [17, 18]. Siderophore biosynthesis is also required for the survival of *Histoplasma capsulatum* within human macrophages. Moreover, a *H. capsulatum* mutant deficient in siderophore synthesis shows significantly reduced the virulence in a mouse model of infection thus indicating an essential role in pathogenesis [19].

Overall, various studies indicate the importance of siderophore biosynthesis and transport in the physiology and virulence of fungi. Therefore, in this review, we summarized what is known about siderophore biosynthesis and transport in selected fungal pathogens to provide an overview of common and unique features and characteristics of the systems. Siderophore biosynthesis and transport in the non-pathogenic fungi, *S. cerevisiae* and *S. chizosaccharomyces pombe*, are also discussed as valuable models.

## Siderophore Biosynthesis and Transport in Model Fungi

### Saccharomyces cerevisiae

*S. cerevisiae* is unable to synthesize its own siderophore but can utilize xenosiderophores produced by other microorganisms via specific transporters. Four siderophore transporters, Arn1, Taf1/Arn2, Sit1/Arn3, and Enb1/Arn4, have been identified and characterized [20-23]. Arn1 is responsible for the uptake of ferrirubin, ferrirhodin, and ferrichrome A, siderophores which contain anhydromevalonyl residues linked to Nd-ornithine (ARN) [22]. Homologs of Arn1 were found in *S. pombe* and *Candida albicans* indicating the protein is conserved in other fungi [24, 25]. Taf1/Arn2 is known to transport triacetylfusarinine [20], and the substrate of Sit1/Arn3 is ferrioxamine B [23]. Unlike other siderophore transporters, which mainly transport hydroxamate siderophores, Enb1/Arn4 facilitates the uptake of the catecholate-type siderophore enterobactin [21]. Furthermore, it was noted that a higher concentration of enterobactin, over 50 μM, inhibits the growth of *S. cerevisiae* because of its strong binding affinity to other essential metals or an unknown activity that interferes with metabolism in the fungus.

The mechanisms by which siderophore transporters take up their substrate and deliver iron into the cytosol has been investigated [26, 27]. In the case of Arn1, two ferrichrome binding sites exist on the surface of the protein. Extracellular ferrichrome is endocytosed via fluid-phase endocytosis and bound to the high-affinity binding site of Arn1 at the endosomal compartment causing a conformational change of the protein and subsequently triggering relocalization of the protein to the plasma membrane. Once Arn1 is located at the plasma membrane, the second molecule of ferrichrome binds to the low-affinity binding site of the protein, which triggers a second conformational change leading to rapid endocytosis and internalization of the ferrichrome-bound Arn1 to cytosol. After endocytosis, intact ferrichrome is eventually dissociated from Arn1, degraded, and iron is released. At the same time, Arn1 is recycled [26, 27]. In this context, observations with the intact holo-form of ferrichrome suggest an iron storage role for the siderophore [27]. Apart from Arn1, the involvement of substrate and ubiquitination-dependent degradation has been proposed for intracellular trafficking of Sit1/Arn3 between the plasma membrane and the vacuole [28]. Moreover, Aft1, a transcriptional activator of the iron regulon in *S. cerevisiae*, directly binds to Sit1/Arn3 and influences the ubiquitination of the transporter [29].

In *S. cerevisiae*, paralogous transcription factors, Aft1 and Aft2 regulate the transcription of genes involved in iron uptake, metabolism and homeostasis (so called the iron regulon) [30, 31]. Although some downstream target genes of these two transcriptional activators overlap, Aft1 localizes in nucleus under iron depletion and primarily activates transcription of genes involved in iron acquisition including siderophore transporters. In contrast, Aft2 activates expression of genes required for intracellular iron utilization [32, 33]. In the high-iron condition, Aft1 and Aft2 migrate to the cytosol and their activity becomes limited. Another transcriptional factor, Yap5, activates genes involved in maintaining iron homeostasis including *CCC1*, which encodes a vacuolar iron importer to store iron within the compartment [33, 34]. Additionally, post-transcriptional regulation controls the expression of genes in the iron regulon. Specifically, Cth1 and its paralog Cth2 are mRNA-binding proteins that regulate degradation of mRNAs encoding functions involved in iron-required metabolism such as respiration, heme biosynthesis and iron-sulfur cluster biogenesis. The expression of genes for Cth1 and Cth2 is induced by Aft1 and Aft2 upon iron deficiency and this suggests that the Cth1 and Cth2 proteins play co-regulatory roles along with the Aft1 and Aft2 proteins [35].

Aft1 directly binds to the iron responsive element (FeRE) consensus sequence, PyPuCACCCPu, of genes involved in iron transport and metabolism [36]. Among the siderophore transporters in *S. cerevisiae*, the *ARN2* genes contains a FeRE in its promoter region and was shown to be direct binding target of Aft1 [37]. Furthermore, as mentioned above, Aft1 physically interacts with Sit1/Arn3 and inhibits degradation of the protein, and increases localization and activity of the protein [29, 38]. In addition to Aft1 and Aft2, other regulatory proteins, Tup1, Cti6, and Ssn6, influence the transcription of *ARN1* and *ARN2* [39, 40].

Apart from Arn siderophore transporters, cell wall proteins are also involved in siderophore uptake and utilization in *S. cerevisiae*. The yeast cell wall is composed of b-glucans, mannoproteins, and chitin, and extracellular compounds including siderophore must penetrate the cell wall structure to gain access to siderophore uptake systems. Genes for three cell wall proteins named Fit1 (facilitator of iron transport), Fit2, and Fit3 were identified as direct regulatory targets of Aft1. Deletion of *FIT* genes results in reduction of ferroxamine B transport suggesting involvement in siderophore uptake [41]. Moreover, a later study using yeast two hybrid screening showed that Sed1, a cell wall mannoprotein involved in cell wall integrity, binds to Arn3 and mediates transport of ferroxamine B in *S. cerevisiae* [42].

### Schizosaccharomyces pombe

*S. pombe* is a fission yeast and is considered as another model unicellular organism, especially for studying the eukaryotic cell cycle, gene structures, and chromatin dynamics [43]. *S. pombe* produces the hydroxamate-type siderophore ferrichrome which mainly accumulates intracellularly, although a small portion is secreted. In the iron-depleted condition, the desferri-form of ferrichrome was observed in the *S. pombe* cells while the iron-bound form was found in the high-iron condition. These observations suggest that ferrichrome acts as an iron storage molecule in *S. pombe* [44]. Three genes involved in ferrichrome biosynthesis have been identified in *S. pombe*. The *sib2* gene, an ortholog of *sid1* and *sidA* in *U. maydis* and *A. nidulans*, respectively, encodes ornithine *N*^5^-oxygenase [45]. The *sib1* gene encodes nonribosomal peptide synthetase and homologous genes encode Sid2 and SidC in *U. maydis* and *A. nidulans*, respectively [46]. Moreover, *sib3* encoding a putative N-transacetylase was identified by ferrichrome-mediated cross-feeding experiments using a *S. cerevisiae* mutant lacking siderophore transporters. Sib3 likely catalyzes acetylation of *N*^5^-hydroxyornithine to produce *N*^5^-acetyl-*N*^5^-hydroxyornithine, which subsequently combines with three glycine residues to form ferrichrome [47].

*S. pombe* possesses the siderophore transporters *str1*, *str2*, and *str3*, which are homologous to *S. cerevisiae* Arn1- 4. The *str1* gene was found as a downstream gene of the GATA-type iron regulatory protein Fep1. Indeed, the 5'-upstream sequence of *str1* contains a single GATA element that is a Fep1 binding site, and transcription of the gene was negatively regulated by Fep1. Furthermore, a complementation test using the *S. cerevisiae* mutant lacking *ARN1* revealed that *str1* is a ferrichrome-specific siderophore transporter [48]. Similar to *str1*, the promoter regions of *str2* and *str3* contain the GATA element, and their expression is also negatively regulated by Fep1 [48].

To date, little information is available on the role of siderophores in the physiology of *S. pombe* except for a contribution of ferrichrome biosynthesis and transport to spore germination. The mutants lacking *sib1* and *sib2* were deficient in spore germination, a phenotype that was restored with exogenously added ferrichrome suggesting the siderophore biosynthesis is essential for the process. However, spore germination no longer took place in a triple mutant, *sib1Δ*, *sib2Δ*, *str1Δ*, although exogenously added ferrichrome was present confirming that *str1* is responsible for ferrichrome uptake. These results indicate that siderophore biosynthesis and transport, and the role of *str1* are critical in the process of spore germination and that *str2* and *str3* were dispensable [49].

## Siderophore Biosynthesis and Transport in Plant Pathogenic Fungi

### Ustilago maydis

*U. maydis* is a phytopathogenic basidiomycete fungus that is mainly parasitic on maize and that causes a smut disease resulting in serious economic losses [50]. Two hydroxamate-type siderophores, ferrichrome and ferrichrome A, are produced by *U. maydis* cells grown in an iron-depleted medium. Ferrichrome is mainly found intracellularly, and is constitutively produced independent of the iron concentration in the medium. In contrast, extracellular ferrichrome was observed only in the culture supernatant of the *U. maydis* cells grown in iron-depleted medium. Ferrichrome A was mainly found extracellularly when *U. maydis* was cultured under the iron-deficient conditions, although a small amount of the siderophore was observed within the fungal cells after an extended growth period [51].

A study utilizing cross-feeding experiments with a non-enterobactin-producing *Salmonella typhimurium* mutant strain and random mutagenesis of *U. maydis* identified the *sid1* gene encoding ornithine-*N*^5^-oxygenase that catalyzes hydroxylation of L-ornithine to *N*^5^-hydroxyornithine; this is the first step in ferrichrome and ferrichrome A biosynthesis [52]. The *U. maydis* mutant lacking *sid1* showed virulence similar to the wild-type in a maize infection model indicating that siderophore biosynthesis is not essential to the pathogenesis of the fungus [53]. Subsequently, the *sid2* gene encoding nonribosomal peptide synthetase was identified and genetically analyzed [54]. Iron restriction caused increased transcript levels of *sid1* and *sid2*, and the GATA-type iron regulatory protein Urbs1 negatively regulates expression of both genes [53, 54]. Additional genes involved in ferrichrome A biosynthesis were identified via microarray analysis to search for regulatory targets of *urbs1*. These genes include *fer3*, *fer4*, and *fer5* encoding a non-ribosomal peptide synthetase, an enoyl-coenzyme A (CoA)-hydratase, and an acylase, respectively. The *hcs1* gene which encodes a hydroxymethyl glutaryl (HMG)-CoA synthase was also identified and is responsible for producing HMG-CoA, a precursor of ferrichrome A [55]. Interestingly, in *S. cerevisiae*, the HMG-CoA synthase Erg13 is responsible for ergosterol biosynthesis and is involved in cell wall and membrane generation [56]. Repression of HMG-CoA production using a conditional promoter caused a swollen morphology and defect in cell separation in *U. maydis* [55]. A transcriptome analysis to search for downstream target genes of the protein kinase A (PKA) catalytic subunit Adr1 also revealed that the expression of *sid1* and *sid2* are regulated by PKA. Moreover, a novel gene, *fer3*, was identified that encodes an ortholog of *A. nidulans* siderophore peptide synthetase SidC, may be involved in ferrichrome biosynthesis [57]. The same study also identified *fer7* encoding a putative siderophore transporter that shows high similarity to *S. pombe* Str3 and *S. cerevisiae* Sit1 [57]. Apart from *fer7*, no additional studies have been carried out to identify and characterize siderophore transporters in *U. maydis*.

### Magnaporthe grisea

The phytopathogen *M. grisea* is the causative agent of blast disease in rice, wheat and barley and causes significant crop losses throughout the world. Several siderophores have been identified in *M. grisea* including ferricrocin, coprogen, coprogen B, 2-*N*-methylcoprogen, and 2-*N*-methylcoprogen B. Among these, ferricrocin was mainly found intracellularly while the others were secreted [46, 58]. A search for genes responsible for siderophore biosynthesis in the genome of *M. grisea* resulted in the identification of *SSM1* and *OMO1*, which are orthologous to *sid2* of *U. maydis* encoding a non-ribosomal peptide synthetase and *sidA* of *A. fumigatus* encoding ornithine-*N*^5^-oxygenase, respectively. Expression analysis showed that transcript levels of *SSM1* were not affected by iron availability, while increased levels of *OMO1* transcripts were observed in cells grown in the iron-depleted medium compared with cells grown in the presence of iron. A biochemical assay with the mutant lacking *SSM1* revealed that the gene is required for intracellular ferricrocin biosynthesis while the production of extracellular siderophores, coprogen, and coprogen derivatives, was not affected. Moreover, an *ssm1Δ* mutant displayed reduced virulence in detached rice leaf assays and decreased ability to penetrate the onion epidermal cell surface suggesting that intracellular ferrichrome biosynthesis plays a critical role in the pathogenesis of *M. grisea* [59]. Subsequently, another gene, *SSM2*, encoding a non-ribosomal peptide synthetase was found in the genome of *M. grisea*. The mutant lacking *SSM2* no longer synthesizes and secretes coprogen and its derivates while ferricrocin biosynthesis was not altered indicating the Ssm2 protein is required specifically for all coprogen-type siderophores. Other phenotypes including reduced growth rate, production of fewer conidia, and increased sensitivity to oxidative stress were observed with the *ssm2Δ* mutant [60]. Deletion of *OMO1* no longer produced any type of siderophore in *M. grisea* suggesting that the ornithine-*N*^5^-oxygenase encoded by the gene is required for both ferricrocin and coprogen biosynthesis. Reduced conidiation, a lower growth rate, and increased sensitivity to oxidative stress were also observed for the *omo1Δ* mutant indicating multiple roles of siderophore biosynthesis in *M. grisea*. Interestingly, the *ssm2Δ* mutant was as virulent as the wild type in detached rice leaves and whole spray-inoculated plants suggesting that *M. grisea* uses siderophore-independent iron uptake during infection [60]. To date, no study has been performed for the identification and characterization of siderophore transporters in *M. grisea*.

## Siderophore Biosynthesis and Transport in Fungal Pathogens of Humans

### Aspergillus fumigatus

*A. fumigatus* is a ubiquitous and saprophytic fungus that causes infections mainly in immunocompromised patients [61]. In fact, *A. fumigatus* is recognized as the major cause of invasive aspergillosis, which has a high mortality rate of 60-90% [62, 63]. An early study showed that *A. fumigatus* produces siderophores of the hydroxamate family, including fusarinine C, triacetylfusarinine C, ferricrocin, and hydroxyferricrocin [64]. Fusarinine C and triacetylfusarinine C are extracellular siderophores for iron uptake, while ferricrocin and hydroxyferricrocin are intracellular siderophores for hyphal and conidial iron storage, respectively [65, 66].

Genes responsible for siderophore biosynthesis have been identified and their functions characterized [65]. The *sidA* gene encodes ornithine *N*^5^-oxygenase, which converts ornithine to *N*^5^-hydroxyornithine and is required for the first step of the siderophore biosynthesis pathway. A mutant lacking *sidA* is unable to synthesize the siderophores triacetylfusarinine C and ferricrocin, and displays a growth deficiency in the low-iron condition. Moreover, the *sidAΔ* strain is not able to grow in the media containing human serum likely because of loss of ability to remove iron from human transferrin. The mutant is completely avirulent in a mouse model of invasive aspergillosis suggesting siderophore biosynthesis is required for the virulence of *A. fumigatus* [67].

The next step in the biosynthesis of siderophores, especially ferricrocin and hydroxyferricrocin, is mediated by SidL, which is *N*^6^-hydroxylysine:acetyl-CoA-*N*^6^-transacetylase that catalyzes acetylation of *N*^5^-hydroxyornithine to *N*^5^-acetyl-*N*^5^-hydroxyornithine, and by a nonribosomal peptide synthetase SidC [66, 68]. Interestingly, the expression of SidL was not affected by extracellular iron levels nor controlled by the GATA-type iron regulatory repressor SreA. Furthermore, the strain lacking SidL displays the decreased production and the reduced size of conidia, and increased sensitivity to oxidative stress confirming ferricrocin and hydroxyferricrocin play critical roles in intracellular iron homeostasis and conidiation [68]. Synthesis of the extracelllualr siderophores, fusarinine C and triacetylfusarinine C, is mediated by SidF, which is a *N*^5^-hydroxyornithine:anhydromevalonyl coenzyme A-*N*^5^-transacylase that transfers cis-anhydromevalonyl-CoA, derived from mevalonate through CoA-ligation and dehydration catalyzed by SidI and SidH, respectively, to *N*^5^-hydroxyornithine [69]. SidD is a nonribosomal peptide synthetase and is responsible for the biosynthesis of fusarinine C. Subsequently, triacetylfusarinine C is produced by SidG [66]. Additionally, a global transcriptional analysis revealed that the mutant lacking the iron regulatory transcriptional HapX showed significantly decreased transcript levels of *sidG* in parallel with the reduced production triacetylfusarinine C, but not fusarinine C. This result indicated that *sidG* in the siderophore biosynthetic pathway is the only gene regulated by HapX in *A. fumigatus* [70].

Deletion of the genes responsible for either SidF and SidD for the triacetylfusarinine C and ferricrocin biosynthesis pathway, or SidC for the ferricrocin and hydroxyferricrocin biosynthesis pathway only caused attenuated virulence in neutropenic mice, while the *sidAΔ* strain displayed completely abolished virulence as mentioned above. These results suggest that both extra- and intracellular siderophores are critical for the virulence of *A. fumigatus* [66]. Furthermore, a study using murine alveolar macrophages showed that killing rates for the mutant strains lacking *sidA* and *sidF* were significantly reduced, and mutants lacking the genes encoding SidF or SidD showed a significantly decreased hyphal length and the growth rate extracellularly and within murine macrophages. These results indicate the crucial roles of siderophore biosynthesis in survival inside host immune cells [17].

*A. fumigatus* is known to have at least seven genes encoding siderophore iron transporters including MirB, MirC, MirD, Sit1, and Sit2 [71, 72]. Among them, MirB, MirD, Sit1, and Sit2 are localized at the plasma membrane while MirC was localized intracellularly, likely in vacuole-like compartment [73]. *A. fumigatus* is not able to synthesize ferrichrome and ferrioxamine B. However, Sit1 is responsible for the uptake of both xenosiderophores while Sit2 is required only for ferroxamine B uptake. Sit1 and Sit2 influenced conidial killing activity although no difference was noted between strains lacking *sit1* or *sit2* and the wild type for mouse survival [74]. The roles of MirA and its paralogues MirB and MirC were initially investigated in *A. nidulans*. MirA and MirB in *A. nidulans* are highly similar to *S. cerevisiae* Arn2 and Arn4 and function as enterobactin and triacetylfusarinine C transporters, respectively [75, 76]. No information on the role of MirC is available [75]. In *A. fumigatus*, MirB and MirD are responsible for the uptake of triacetylfusarinine C and fusarinine C, respectively [18, 77], and MirC is involved in regulating the levels of intracellular siderophore (in particular ferricrocin). The contribution of MirC in the virulence of *A. fumigatus* using the *Galleria mellonella* infection model was demonstrated as well [73]. Moreover, the mutant lacking the gene encoding MirB, but not MirD, reduced virulence in a murine aspergillosis model suggesting the importance of the functions of MirB in the host [18]. The expression of all of the siderophore transporters was responsive to iron levels in the environment. Transcript levels of MirB, MirC, and Sit1 were reduced in the absence of SreA and the mutant lacking HapX showed decreased transcript levels of MirB suggesting positive regulatory roles of both iron regulatory transcription factors on siderophore transport in *A. fumigatus* [70, 78]. Various studies have used sequence information and functional characteristics of siderophore transporters in *A. fumigatus* to investigate homologous systems in other fungi (Table 2).

### Candida albicans

*C. albicans* does not possess a siderophore biosynthesis pathway but is able to utilize xenosiderophores. Only a single siderophore transporter Sit1/Arn1 has been identified and characterized, and the hydroxamate-type siderophores such as ferrichrome, ferricrocin, ferrichrysin, and ferrirubin are substrates. However, a mutant lacking *SIT1*/*ARN1* showed normal growth in the presence of ferrioxamine B and E suggesting the presence of a yet unknown pathway responsible for ferroxamine utilization [79-81]. A role for Sit1/Arn1 in virulence of *C. albicans* has been suggested. Initially, it was found that expression of *SIT1*/*ARN1* was up-regulated in the cells grown in the iron-depleted condition as well as in hyphal cells implying possible association of the role of siderophore transporter with virulence [81]. However, *SIT1*/*ARN1* is dispensable in the murine model of *Candida* dissemination although it was shown to be required for virulence in an *in vitro* model of oral candidiasis [79]. Homologs of Sit1 have been identified and characterized in the non-albicans *Candida* species including *C. glabrata*. For example, Sit1 is the only siderophore transporter in *C. glabrata* and is required for survival within macrophages [82].

Global expression analysis showed that Sfu1, a GATA-type transcription factor, regulates the expression of genes involved in iron uptake and metabolism in *C. albicans*, and one of the downstream target gene was *SIT1*/*ARN1*. Indeed, the *C. albicans* mutant lacking *SFU1* displayed significantly up-regulated transcript levels of *SIT1*/*ARN1* [83]. Moreover, a later study using transcriptome analysis and chromatin immunoprecipitation revealed that, in addition to direct and negative regulation by Sfu1, Sef1 (Zn_2_Cys_6_ DNA-binding protein) directly and positively regulates transcription of *SIT1*/*ARN1* [84]. Interestingly, Sef1 not only positively regulates siderophore uptake but also controls other genes responsible for iron transport and homeostasis, and is required for virulence in a murine bloodstream infection model [84].

In addition to the transport of siderophores for its own growth, *C. albicans* influences siderophore utilization of other microorganisms in the situation of polymicrobial infection. For example, *C. albicans* and *Pseudomonas aeruginosa* often coinfect and colocalize various body sites of humans such as the gut, lung, burn wounds, and the genitourinary tract. Interestingly, when *C. albicans* was coinfected and colocalized with *P. aeruginosa*, the fungus interfered with production of the bacterial siderophores pyochelin and pyoverdine and increased the survival of the mammalian host [85].

### Cryptococcus neoformans

*Cryptococcus neoformans* is the causative agent of fungal meningitis in immunocompromised patients including individuals living with HIV. Remarkably, cryptococcal disease accounts for one in five deaths of AIDS patients globally indicating the clinical importance of this life-threatening fungus [86, 87]. As in other fungal pathogens, iron acquisition and homeostasis are crucial for the virulence of *C. neoformans*. Regulation of the expression of key virulence factors such as capsule formation and melanin synthesis by iron availability contributes to disease [88].

*C. neoformans* is unable to synthesize siderophores but can utilize xenosiderophores produced from other microbes as an iron source. A siderophore transporter Sit1, which is responsible for ferrioxamine B uptake, was characterized in *C. neoformans* [89]. Sit1 is not only required for siderophore transport but also influences melanin formation and cell wall integrity implying an additional physiological function of the protein. However, the mutants lacking *SIT1* still caused the wild-type level of virulence in a murine model of cryptococcosis suggesting that *SIT1* was dispensable for disease [89]. In addition to Sit1, at least five more paralogs of Sit1, Sit2-Sit6, were found in the genome of *C. neoformans*, but their functions and roles in physiology and virulence in the fungus have not been demonstrated yet. A GATA-type transcription factor Cir1, which is a major iron regulatory protein in *C. neoformans*, binds to the promoter region of *SIT1* and *SIT2*, and positively regulates their expression under the low-depleted condition while the protein binds to the promoter region of *SIT4* and *SIT6* and represses gene expression under the high-iron condition [90]. Another major iron regulatory transcription factor HapX, which is a regulatory subunit of the CCAAT-binding complex, binds to the promoter region of SIT3 under the low-iron condition and activates expression of the gene [90].

### Histoplasma capsulatum

*H. capsulatum* is a thermally dimorphic fungus that causes histoplasmosis, a disease of both immunocompetent and immunocompromised individuals such as cancer or AIDS patients [91]. *H. capsulatum* produces at least three hydroxamate siderophores, dimerum acid, coprogen B, and fusarinine [92]. Global transcription analysis to identify differentially expressed genes in *H. capsulatum* grown in low-iron versus high-iron conditions revealed that a set of genes, *SID1*, *SID3*, *SID4*, *NPS1*, *OXR1*, *MFS1*, and *ABC1*, encoding an ornithine-*N*^5^-oxygenase, an acetylase, an acid co-A ligase, a non-ribosomal peptide synthase, an oxidoreductase, a major facilitator superfamily transporter, and an ATP-binding cassette transporter, respectively, were highly induced by iron depletion [19]. The strain lacking *SID1* grew poorly and did not produce siderophore. Furthermore, the *sid1Δ* strain showed a growth deficiency in macrophages derived from the murine bone marrow and was attenuated in the mouse model of the infection indicating that siderophore biosynthesis and transport are critical in the virulence of *H. capsulatum* [19, 93]. Similar to other pathogenic fungi, *H. capsulatum* possesses a GATA-type transcription factor, Sre1, which regulates iron-dependent transcription of siderophore biosynthesis genes [94, 95].

An involvement of peroxisomes, organelles that function in multiple metabolic pathways including the glyoxylate cycle, fatty acid b-oxidation, and metabolism of reactive oxygen species, in siderophore synthesis was reported for *H. capsulatum*. The *H. capsulatum* Sid1 and Sid3 proteins contain a PTS1 tripeptide motif for localization in peroxisomes [96, 97]. Moreover, deletion of genes encoding peroxisomal proteins such as Pex5, Pex10, and Pex33 resulted in a significant decrease in siderophore production and deficient growth in the low-iron condition [97].

## Conclusion

**Fig. 1:**
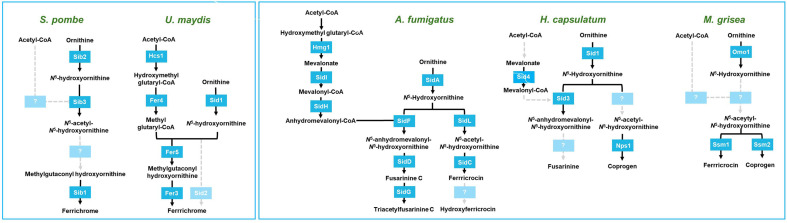
Siderophore synthesis pathways identified in representative fungal species. Dotted lines indicate the pathways and enzymes not identified yet. *S. pombe* and *U. maydis* produce ferrichrome while *A. fumigatus*, *H. capsulatum*, and *M. grisea* synthesize fusarinine, ferricrocin, and coprogen siderophores.

Accumulating experimental evidence indicates that siderophore biosynthesis and transport play crucial roles in growth, development, stress responses (*e.g.*, to oxidative stress) and, most importantly, survival and virulence of fungal pathogens in the host environment. Detailed studies have revealed how *S. cerevisiae* transports and utilizes siderophores as well as the regulation of siderophore uptake in response to iron levels in the environment. The siderophore transport system in *S. cerevisiae* is a valuable model for other fungi, and indeed several fungi including *C. albicans* and *C. neoformans* were shown to possess homologs of the *S. cerevisiae* Arn siderophore transporters. Synthesis of fungal siderophores is best characterized in *U. maydis* and *A. fumigatus*. *U. maydis* mainly produces ferrichrome siderophores while *A. fumigatus* produces fusarinines and ferricrocin type siderophores, which are extracellular and intracellular siderophores, respectively (Fig. 1). Moreover, the contribution of siderophore biosynthesis, transport, and its regulation in the mammalian host has been clearly demonstrated in *A. fumigatus*.

Iron assimilation using siderophores is of particular interest in human fungal pathogens not only because of their involvement in virulence but also because of their value for the development of novel antifungal strategies and other possible therapeutic applications. For example, much attention has been paid to generating siderophore-antifungal drug conjugates, siderophore-fluorophore conjugates, and labeling of siderophores with radionuclides to develop novel fungal diagnostic and therapeutic tools [98-100]. Further studies are needed to understand siderophore biosynthesis, transport (excretion and uptake), and the mechanisms of iron release from iron-bound siderophore upon internalization. Additionally, a broader survey of siderophore biosynthesis and transport machinery is needed to understand contributions to survival and virulence strategies in many other yet unexplored fungi, including emerging fungal pathogens of humans such as *Candida auris*.

## Figures and Tables

**Table 1:** Five main types of siderophores identified from bacteria and fungi.

Type	Siderophores	Reference
Hydroxamate	Coprogen, Coprogen B, Fusarinine, Ferrichrome, Ferrichrome A, Ferricrocin, Ferrirubin, Ferrirhodin, Desferrioxamine B, Rhodotorulic acid, Fusarinine C, Triacetylfusarinine C	[58, 72, 101-104]
Carboxylate	Vibrioferrin, Staphyloferrin A, Rhizoferrin, Achromobactin, Citrate	[72, 105-107]
Catecholate	Enterobactin, Salmochelin, Chrysobactin	[108-110]
Phenolate	Yersiniabactin, Pyochelin	[111, 112]
Mixed class	Pyoverdine, Mycobactin, Aerobactin, Anguibactin	[113-116]

**Table 2:** Siderophore transporters of *Aspergillus fumigatus* and their homologs in other fungi.